# Heterogeneous Participant Recruitment for Comprehensive Vehicle Sensing

**DOI:** 10.1371/journal.pone.0138898

**Published:** 2015-09-25

**Authors:** Yazhi Liu, Xiong Li

**Affiliations:** 1 College of Information, North China University of Science and Technology, Tangshan 063009, China; 2 School of Computer Science and Engineering, Hunan University of Science and Technology, Xiangtan 411201, China; Nanyang Technological University, SINGAPORE

## Abstract

Widely distributed mobile vehicles wherein various sensing devices and wireless communication interfaces are installed bring vehicular participatory sensing into practice. However, the heterogeneity of vehicles in terms of sensing capability and mobility, and the participants’ expectations on the incentives blackmake the collection of comprehensive sensing data a challenging task. A sensing data quality-oriented optimal heterogeneous participant recruitment strategy is proposed in this paper for vehicular participatory sensing. In the proposed strategy, the differences between the sensing data requirements and the collected sensing data are modeled. An optimization formula is established to model the optimal participant recruitment problem, and a participant utility analysis scheme is built based on the sensing and mobility features of vehicles. Besides, a greedy algorithm is then designed according to the utility of vehicles to recruit the most efficient vehicles with a limited total incentive budget. Real trace-driven simulations show that the proposed strategy can collect 85.4% of available sensing data with 34% incentive budget.

## 1 Introduction

Different from the work mode of traditional wireless sensor networks [1, 2], in a participatory sensing system, ordinary citizens are recruited to collect and share sensing data from their surrounding environments through their mobile devices [3]. Based on the collected sensing data, participatory sensing systems are able to provide various novel applications to the public [4], such as real-time air quality report [5] and road traffic monitoring [6, 7]. An increasing number of vehicles are being installed with various sensing devices and mobile communication interfaces. Therefore, an increasing number of mobile vehicles are able to collect and share various kinds of sensing data for urban environments. The extensive distribution of these vehicles in urban areas brings vehicular participatory sensing into practice.

A vehicle participatory sensing system is composed of sensing vehicles, wireless networks, and sensing data centers. Sensing data are collected by sensing vehicles and then transmitted to a sensing data center via wireless networks. The application of vehicular participatory sensing to transportation can generate many advantages. First, it can lower the cost of collecting sensing data because vehicles are equipped with several sensors. Second, compared with the single-vehicle sensing system, in the vehicular participatory sensing system, sensing data are collaboratively collected by multiple vehicles. As a result, the vehicular participatory sensing system can collect a large amount of comprehensive sensing data. Third, owing to the collaboration between vehicles and sensing data sharing, the vehicular participatory sensing system can provide many novel applications to the field of transportation and other areas.

Collection and transmission of sensing data consume a considerable amount of vehicle resources. Furthermore, the uploaded sensing data may leak the private information of the participants [8]. As a result, vehicle recruitment and the deployment of the vehicular participatory sensing system are restricted by resource consumption or privacy disclosure. With regard to the fact that participating in the collection of sensing data may incur real monetary costs on the part of the participants (e.g., data traffic fee), Ra et al. [9] pointed out that an incentive mechanism is necessary for a participatory sensing system to motivate more participants to contribute their sensing data and maintain the sustainability of the system. Reference [10] shows that individuals are willing to participate in sensing systems when they are paid as little as 25 cents.

Vehicular participatory sensing systems should thus motivate vehicle drivers to participate in the collection of sensing data by providing the drivers certain rewards. However, in a specific vehicular participatory sensing system, the total incentive budget is usually limited. Consequently, the number of vehicles that could be recruited to participate in sensing data collection is also limited. The quality of the sensing data collected by a vehicular participatory sensing system is thus constrained by the total incentive budget provided by the system.

The sensing interfaces of vehicles and the types of sensing data that can be collected by vehicles are different [11] because vehicles are manufactured by different companies with no uniform hardware/software standards. Vehicles are driven by different people, and the vehicle trajectories and expectations of people on the incentives also differ. Therefore, the participants in a vehicular participatory sensing system are heterogeneous.

The purpose of a participatory sensing system is basically to collect comprehensive sensing data that encompass all the spatial-temporal dimensions of the target sensing area. The incentive budget of participatory sensing systems is limited, and as a result, the number of participants that could be recruited is also limited. The heterogeneity of the participants in sensing interfaces, the trajectories, and the expectations on the incentives render the collection of comprehensive sensing data challenging in vehicular participatory sensing systems. Therefore, how to recruit appropriate participants from candidates to collect satisfactory sensing data for a target area with a limited incentive budget is a crucial issue in vehicular participatory sensing systems.

To address the abovementioned challenges, a heterogeneous participant recruitment strategy is designed in this study to select a set of participants who can collect desirable sensing data in both temporal and spatial dimensions under a constrained budget. blackThe major contributions of this study include the following.

- A participant utility analysis scheme is established based on the sensing and mobility features of the participants. Utility is utilized to evaluate the incentive efficiency of the sensing participants based on the ratio of their expected data contributions to their incentive requirements.
- A heterogeneous participant selection strategy is designed to recruit participants. The strategy is based on a greedy algorithm that evaluates the participants’ efficiencies according to their utilities.

The remainder of this paper is organized as follows. Related studies on participatory and vehicle sensing are reviewed in Section 2. Section 3 presents the vehicular participatory sensing system model and the construction of the system-requested sensing data models. The optimal heterogeneous participant recruitment problem is formulated in Section 4. Section 5 presents the design of the optimal heterogeneous participant recruitment (HPR) strategy, which considers the balance between constrained budget and effectiveness of sensing data. The performance of the proposed strategy is evaluated in Section 6 through real trace-driven simulations. Section 7 provides the conclusions.

## 2 Related Work

Participatory sensing [3], also known as mobile crowd sensing [12, 13] and opportunistic sensing [14], is a promising means to collect comprehensive sensing data in urban areas. A large number of novel applications [4] based on participatory sensing have been developed in recent years for environmental protection, road traffic management, medical treatment, and so on.

Comprehensive collection of sensing data is the most important criterion of participatory sensing systems. Sensing data collected by 85 mobile nodes for two months reveal that people are more willing to collect sensing data from areas that have a small population [15]. Reverse auction based dynamic price (RADP) [16] introduced incentive mechanisms to participatory sensing systems. The cheapest sensing data are utilized to increase the total amount of sensing data collected by the participants; as a result, the accuracy of sensing data is improved. However, participants are usually clustered by time and space. Consequently, the collected data are inhomogeneous in time and space. To collect homogeneous sensing data, greedy budgeted maximum coverage (GBMC) [17] maximized not only the amount of collected sensing data but also the coverage of sensing data. ISAM [18] minimized the diversity between collected data and the forecasted model to improve the quality of sensing data. Unlike RADP, GBMC and ISAM investigated the homogeneous space distribution of sensing data. However, none of them considered the time distribution of collected data.

In participatory sensing systems, the crowd mobile participants frequently encounter one another and are thus provided an opportunity to collaborate and provide high-quality sensing services. Collaborative sensing is one of the essential features of participatory sensing systems. Context characteristics are generated from the sensing data collected by multiple nearby participants through the use of the collaborative learning algorithm [19]. The collaborative learning method can significantly improve the learning precision. This learning method is based on the sensing data collected by an individual participant. Mobile Sensor Data Engine (MOSDEN) [20] is a collaborative mobile sensing framework that can operate on smartphones to capture and share sensed data among multiple distributed applications and users. Similarly, a cloud-assisted collaborative sensing method was proposed in [21] to reduce the energy consumption of mobile phone sensing applications.

Participant recruitment is a major challenge in participatory sensing systems because of the diversity of the sensing capabilities of mobile devices and the uncontrollable trajectories of mobile participants. Chien et al. [22] introduced the online task assignment problem in which heterogeneous tasks are assigned to workers with different, unknown skill sets. Reddy et al. [23] developed a selection framework to allow organizers to identify well-suited participants for data collection based on their geographic and temporal availability as well as their habits. Tuncay et al. [24] exploited the stability of user behavior and selected participants based on the fitness of the mobility history profiles of the users. However, none of the previous studies considered both the limited incentive and the heterogeneity of the participants.

The vehicle is a special type of participant in participatory sensing systems. Considering that vehicles are widely distributed in urban areas and equipped with various types of sensors, vehicles provide a natural means to mobile sensing. In early vehicle-based environmental sensing applications, only one vehicle is recruited in the system. One of the novel applications is the monitoring of locations through a vehicle radar [25]. Air quality can also be monitored by a *CO*_2_ sensor installed in a vehicle [26]. Similarly, rainy weather can be monitored by an on-board camera [27]. In [28], a laser scanner and an on-board camera were utilized to update a street view map. However, in these applications, no sensing data are shared among vehicles. This condition constrains the extensive deployment of vehicle sensing-based applications.

A platform was designed in [29] for large-scale vehicle collaborative sensing. However, the algorithm was designed to plan the trajectories of a robot team to collect sensing data within the shortest time. In [30], multiple probe vehicles were utilized to estimate large-scale traffic in an urban environment. Vehicle sensing can also be utilized for safe driving. RWIS [31] recruited probe vehicles equipped with GPS, an anti-lock braking system, and acceleration sensors to quickly detect the conditions of road surfaces according to the side slip force of the vehicles in certain road segments.

## 3 System Model

### 3.1 System Architecture

**Fig 1:**
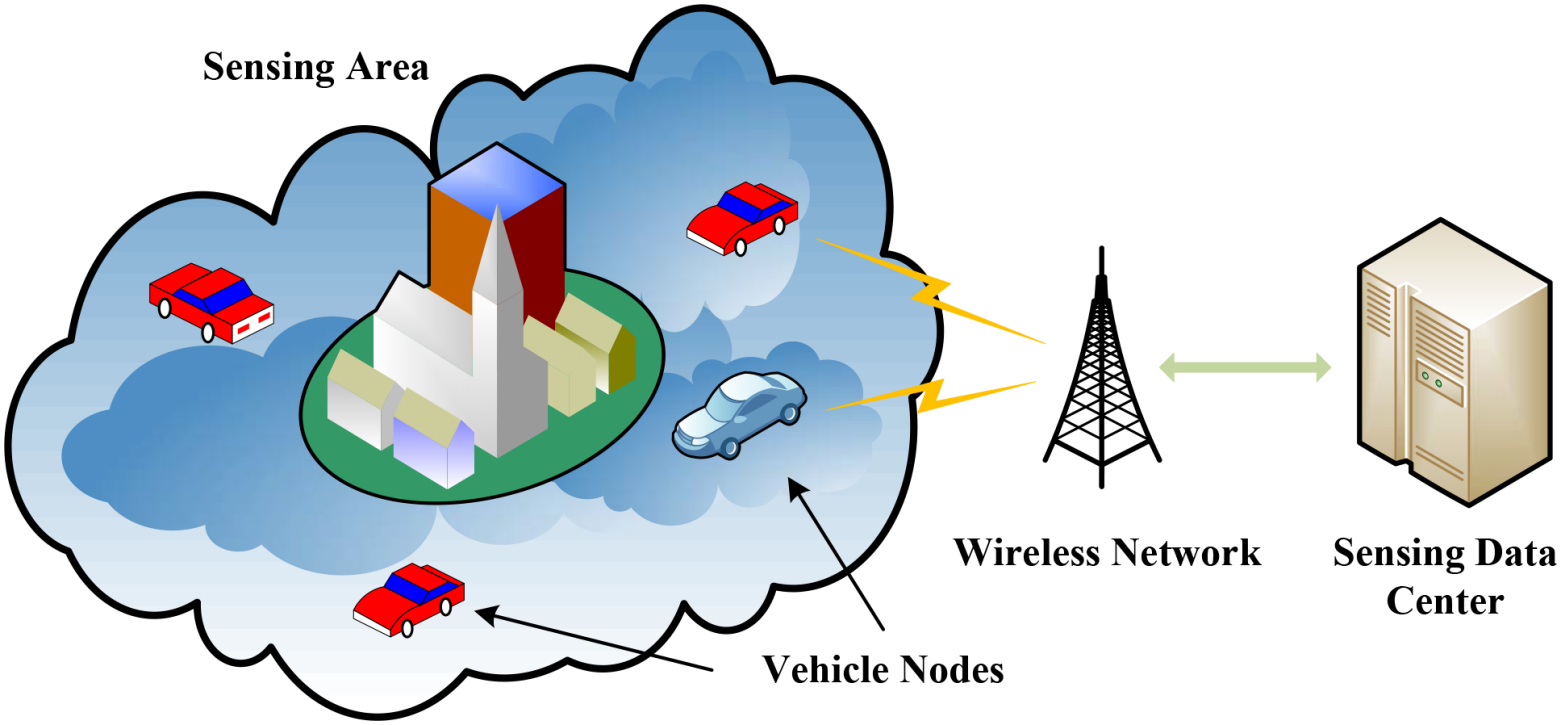
Architecture of vehicular participatory sensing systems.

A vehicular participatory sensing system is composed of sensing data servers, wireless networks, and vehicles (i.e., participants), as shown in Fig 1. Sensing tasks and their detailed information are established in the sensing data server by the system users. The detailed information of a sensing task includes the target sensing area, the sensing time period, and the resolution of the sensing data. The sensing data server recruits participants according to the requirements of the tasks. When a vehicle is recruited as a participant, the sensors embedded in it are turned on to sample the sensing data periodically. All the collected sensing data in combination with the corresponding information of the GPS coordinates are uploaded and stored in the sensing data server via wireless networks. Users of the sensing system can then make a query on sensing data of interest from the sensing data server.

**Fig 2:**
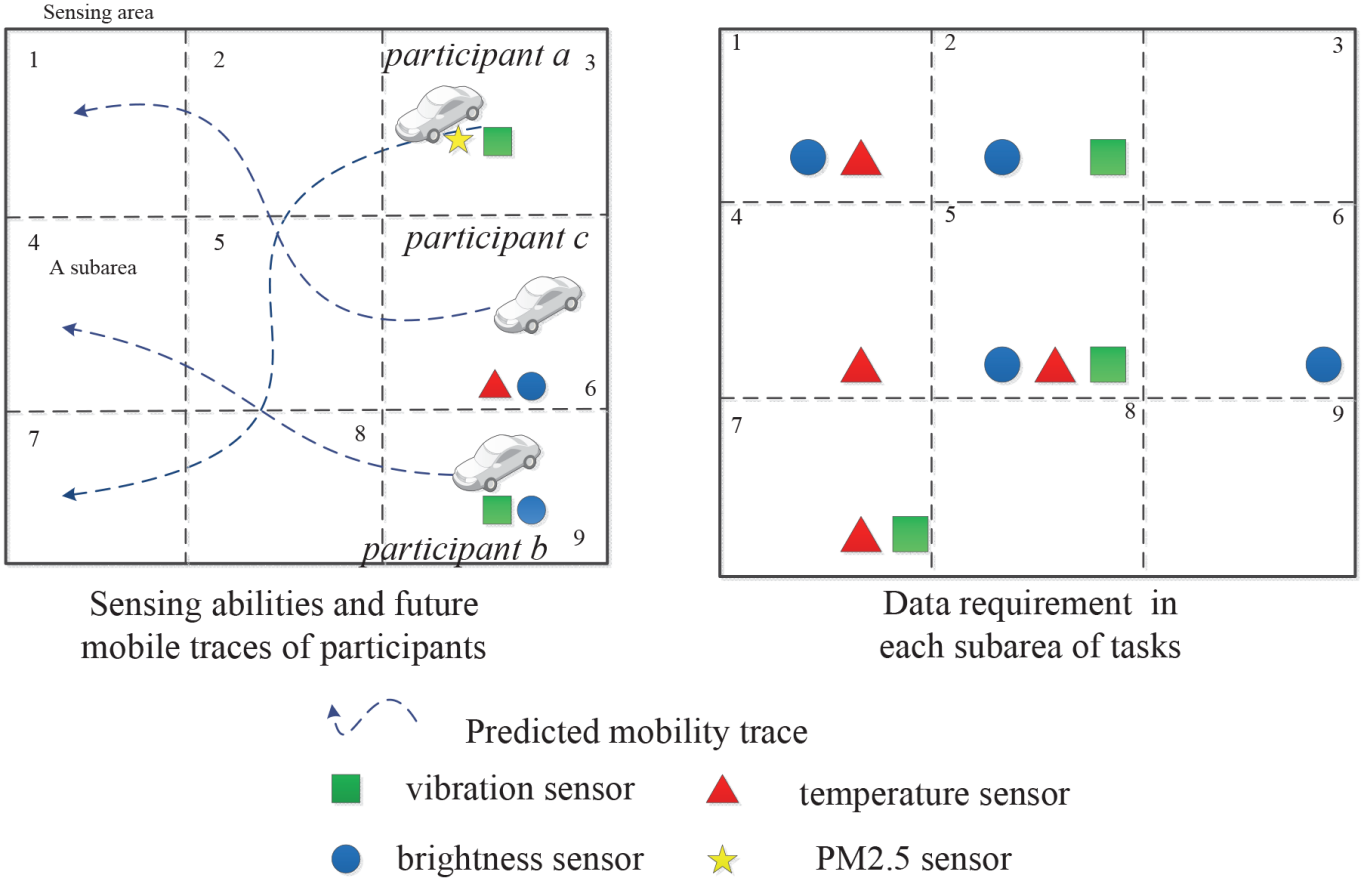
Data collection by different participants for multiple sensing tasks.

Different participant recruitment strategies could result in different effects on fitting the sensing task data requirements. An example of a participant recruitment scenario with multiple sensing tasks is shown in Fig 2. The right part of the figure shows the sensing data requirements in all subareas, and the left part of the figure shows the sensing capabilities of the vehicles and the future mobility traces of all available participants. In this sensing scenario, {*a*, *c*} are obviously more appropriate than {*a*, *b*} to be selected as participants to meet the task requirements. However, in real-world scenarios with a significantly larger number of sensing tasks and available heterogeneous participants, the recruitment of appropriate participants becomes an arduous task.

This paper presents a participant recruitment strategy that can collect the most comprehensive sensing data for all sensing tasks in both temporal and spatial dimensions under limited incentives. The first step in the proposed approach is to formalize the sensing data requirements of the sensing tasks and the sensing data collection expectation of the participating vehicles.

### 3.2 Sensing Data Model

The data required by users and the sensing data that can be collected by the participants are both represented by 2D data matrixes (i.e., temporal and spatial dimensions).

The target sensing area is divided into lattice cells based on the geographical location (Fig 2), and each lattice cell is called a subarea. A subarea is a unit area of the sensing system; the assumption is that sensing data sampled at any point in the subarea can indicate the sensing value of the subarea.

Given that sensing data are usually sensitive to time (e.g., air temperature data collected an hour ago are no longer accurate), sensing data should be sampled periodically in each unit sensing area. The requested sensing data sampling amount indicates how many times of data sampling are necessary in a sensing period in a unit subarea.

The entire sensing area is divided into *l* subareas in the form of grids based on the geographical coordinates (Fig 2). We let *L* = {1,2,⋯, *l*} denote the set of subareas. *i*(*i* ∈ *L*,1 ⩽ *i* ⩽ *l*) denotes one of the subareas. The sensing system runs the participant recruitment algorithm periodically. We suppose that the duty cycle of the system is *T* = *m*, which means that the length of duty cycle *T* is *m* unit times, and let *k*(1 ⩽ *k* ⩽ *m*) denote the *k*th unit time of the system duty cycle. We also suppose that sensing task set *J* = {1,2,⋯, *n*} exists in the sensing area within one system duty cycle and let *j* ∈ *J*,1 ⩽ *j* ⩽ *n* denote one of the tasks in *J*.

We let *M* = {*M*_1_, *M*_2_,⋯,*M*_*N*_} denote the set of all available vehicles, and *N* is the amount of available participants. *C*_*total*_ is the total incentive budget provided by the system for one duty cycle, and *C*_*α*_ denotes the incentive required by participant *α* ∈ *M* if *α* is recruited as a sensing participant during one system duty cycle *T*. We also suppose that set *X* contains the participants recruited from all available participants *M*. We let *ϕ*(*X*) denote the total incentive within a unit time period required by all the recruited participants in *X*. *ϕ*(*X*) can be calculated as

$$\begin{array}{c} \phi (X)=\sum_{\alpha \in X}C_{\alpha } \end{array}$$ (1)

Therefore, the recruitment of *X* would be subjected to *X* ⊂ *M* and *ϕ*(*X*) ⩽ *C*_*total*_.

For a special task *j*, let $R_{ik}^{j}$ be the data requirement in the subarea *i* during the *k*th unit sensing time of the system duty cycle. Then, the data requirement matrix of task *j* can be denoted as,

For specific task *j*, we let $R_{ik}^{j}$ be the data requirement in subarea *i* during the *k*th unit sensing time of the system duty cycle. Then, the data requirement matrix of task *j* can be denoted as **R** ^*j*^ in Eq (2).

$$\begin{array}{c} R^{j}=[\begin{array}{cccc} R_{11}^{j} & R_{12}^{j} & ... & R_{1m}^{j}\\ R_{21}^{j} & R_{22}^{j} & & \\ ... & & ... & \\ R_{l1}^{j} & & & R_{lm}^{j} \end{array}] \end{array}$$ (2)

In the sensing server, when a sensing data sample of task *j* at time *k* in location *i* is reported by a participant, the collected data amount $D_{ik}^{j}\left(X\right)$ would increase by 1. The total data amount of task *j* collected by the participants in *X* could be denoted by the following matrix.

$$\begin{array}{c} D^{j}(X)=[\begin{array}{cccc} D_{11}^{j}\left(X\right) & D_{12}^{j}\left(X\right) & ... & D_{1m}^{j}\left(X\right)\\ D_{21}^{j}\left(X\right) & D_{22}^{j}\left(X\right) & & \\ ... & & ... & \\ D_{l1}^{j}\left(X\right) & & & D_{lm}^{j}\left(X\right) \end{array}] \end{array}$$ (3)

Table 1 presents the list of notations employed in this paper.

**Table 1:** List of notations.

**Notation**	**Description**
*J*	Sensing tasks
*L*	Subareas
*T*	System duty cycle
*C*_*α*_	the incentive required by *α* in one system duty cycle
*C*_*total*_	Total incentives for each system duty cycle
**R** ^*j*^	Data requirements matrix for task *j*
$R_{ik}^{j}$	Data requirements for task *j* in location *i* during time *k*
*M*	All available participants
$S_{\alpha }^{j}$	Sensing ability of a participant *α* to task *j*
*ɛ*	The sensing data sampling interval
$c_{1},c_{2},\ldots ,c_{M_{N_{total}}}$	Incentives demanded by participants for one sampling
*X*	A set of recruited participants
*ϕ*(*X*)	Total incentives required by participants in *X* during one system duty cycle
**D** ^*j*^(*X*)	Collected data matrix for task *j* by *X*
$D_{ik}^{j}(X)$	Collected data amount by *X* of task *j* in location *i* during time *k*
*λ*_1_,*λ*_2_,…,*λ*_*n*_	Weighting coefficient of tasks
**ϴ** ^*j*^(*α*)	The expected data collection by *α* for task *j* in the following system duty cycle
**ψ** ^*j*^	temporary data requirements of task *j*
$p_{i,j}^{t}$	Probability that a node transfers from *L*_*i*_ to *L*_*j*_ in *t*
*U*_*α*_	the sensing utility of *α*

## 4 Optimal Heterogeneous Participant Recruit Mechanism

According to the model defined in Section 3, the problem of collecting the most comprehensive sensing data for all tasks in both temporal and spatial dimensions within incentive constraints is transferred to find set *X* that can minimize the difference between the required and collected data matrixes for all tasks. Given that the Frobenius norm is utilized to measure the spatial length of a matrix, the minimization of the difference between the two matrixes for all tasks can be described by the following multi-objective optimum model.

$$\begin{array}{l} Min\;\left(||R^{1}-D^{1}\left(X\right)|{|}_{F}\right)\\ Min\;\left(||R^{2}-D^{2}\left(X\right)|{|}_{F}\right)\\ \vdots \\ Min\;\left(||R^{n}-D^{n}\left(X\right)|{|}_{F}\right)\\ s.t.:X\subset M\text{,}\\ \;\phi \left(X\right)⩽{\text{C}}_{total} \end{array}$$ (4)

where

$$\begin{array}{c} {∥R^{j}-D^{j}∥}_{F}=\sum_{i=1}^{l}\sum_{k=1}^{m}{\left(R_{ik}^{j}-D_{ik}^{j}(X)\right)}^{2} \end{array}$$ (5)

The Frobenius norm of the difference between the matrixes increases in two situations. When the obtained data amount $D_{ik}^{j}\left(X\right)$ is less than the requested data amount $R_{ik}^{j}$, the Frobenius norm decreases with the increase in the collected data amount $D_{ik}^{j}\left(X\right)$. When the collected data amount $D_{ik}^{j}\left(X\right)$ is more than the requested data amount $R_{ik}^{j}$, the Frobenius norm decreases with the over-collection of sensing data; this condition increases the energy consumption and bandwidth occupation of the participants.

Considering that the multiple-objective optimization problem is difficult to solve, the problem is first converted into a simple objective optimization one through a weighting method. We let *λ*_1_, *λ*_2_,⋯, *λ*_*n*_ denote the weighting coefficients of different sensing tasks and *λ*_*j*_ be the the significance factor of sensing task *j*. The optimization objective function of Eq (4) can then be transformed into

$$\begin{array}{ccc} f(X) & = & \sum_{j=1}^{n}\lambda_{j}\times {∥R^{j}-D^{j}(X)∥}_{F}\\ & = & \sum_{j=1}^{n}\lambda_{j}\times (\sum_{i=1}^{l}\sum_{k=1}^{m}{\left(R_{ik}^{j}-D_{ik}^{j}(X)\right)}^{2}) \end{array}$$ (6)

To minimize the weighted differences between the sensing data collected by the participants and the data requirements, a set of participants that can minimize the value of *f*(*X*) in Eq (6) is determined as follows:

$$\begin{array}{ccc} Min: & & f(X)\\ s.t.: & & X\subset M,\\ & & \phi (X)⩽C_{total} \end{array}$$ (7)

## 5 Participant Recruitment Algorithm

In this section, the utility of the participants is designed based on their expected contributions to the sensing data. A greedy participant recruitment algorithm is proposed to solve the optimization problem of participant selection.

### 5.1 Utility of the Participants

A participant utility evaluation methodology is established according to the different features of the participant nodes to recruit appropriate participants in the collection of sensing data. The utility of the participants should reflect the amount of the required sensing data a participant can collect in one system duty cycle.

We let **ϴ** ^*j*^(*α*) denote the amount of sensing data that participant *α* can collect in one system duty cycle for task *j*, and **ϴ** ^*j*^(*α*) is a matrix as shown in Eq (8).

$$\begin{array}{c} \Theta^{j}(\alpha )=[\begin{array}{cccc} \theta_{11}^{j}(\alpha ) & \theta_{12}^{j}(\alpha ) & \cdots & \theta_{1m}^{j}(\alpha )\\ \theta_{21}^{j}(\alpha ) & \theta_{22}^{j}(\alpha ) & & \\ \vdots & & \cdots & \\ \theta_{l1}^{j}(\alpha ) & & & \theta_{lm}^{j}(\alpha ) \end{array}] \end{array}$$ (8)

where $\theta_{ik}^{j}\left(\alpha \right)$ denotes the expected sensing data amount that participant *α* can collect from subarea *i* at time *k* for sensing task *j*.

The probability-based method [32] employs historical trace data to calculate the probability of moving from one location to another after a certain period of time. We let $p_{ab}^{t}\left(\alpha \right)$ denote the probability that participant *α* transfers from subarea *a* to subarea *b* after time duration *t*. With the trajectory prediction method proposed in [32], for a certain tuple {*t*, *i*, *j*}, the value of $p_{ab}^{t}\left(\alpha \right)$ can be derived from the history traces of participant *α* as follow.

pabt=Nb,a(t)/Nb,a(t)NaNa (9)

Where, *N*_(_ *b*, *a*)(*t*) is the number of times the vehicle has transferred from subarea *a* to subarea *b* after time duration *t*. *N*_*a*_ is the number of times the vehicle has traveled in subarea *a*.

The sampling cycle of all participants is set to *ɛ* (*ɛ* < 1). Therefore, the number of data samples of a specific task contributed by a participant in a unit sensing time period can be calculated as ⌈1/*ɛ*⌉. We suppose that the initial location of participant *α* is *a*. Then, $\theta_{ik}^{j}\left(\alpha \right)$ can be calculated as

$$\begin{array}{c} \theta_{ik}^{j}\left(\alpha \right)=\sum_{\delta =0}^{\left\lceil 1/\varepsilon \right\rceil }\left(p_{ai}^{k}\cdot S_{\alpha }^{j}\right) \end{array}$$ (10)

where the value of $S_{\alpha }^{j}$ is determined by whether participant *α* can collect sensing data for sensing task *j*, as shown in Eq (11).

$$\begin{array}{c} S_{\alpha }^{j}=\{\begin{array}{cc} 0 & \alpha \;\text{is}\;\text{not}\;\text{able}\;\text{to}\;\text{collect}\;\text{data}\;\text{for}\;\text{task}\;j\\ 1 & \alpha \;\text{is}\;\text{able}\;\text{to}\;\text{collect}\;\text{data}\;\text{for}\;\text{task}\;j \end{array} \end{array}$$ (11)

Thus, if *α* is recruited to participate in sensing data collection, the expected contribution of *α* to the quality of sensing data in the sensing system can be expressed as follows:

$$\begin{array}{ccc} g(\alpha ) & = & \sum_{j=1}^{n}\lambda_{j}\times {∥R^{j}-\Theta^{j}(\alpha )∥}_{F}\\ & = & \sum_{j=1}^{n}\lambda_{j}\times (\sum_{i=1}^{l}\sum_{k=1}^{m}{\left(R_{ik}^{j}-\theta_{ik}^{j}(\alpha )\right)}^{2}) \end{array}$$ (12)

However, the difference between the sensing capabilities of two participants is indeterminate. Therefore, recruiting participants at the same time based on the value of *g*(*α*) in Eq (12) is unreasonable.

For example, we suppose that the sensing capability of participants *α* and *β* are similar. Then, according to Eq (12), the value of *g*(*α*) equals that of *g*(*β*). If the participant recruitment algorithm recruits participants simply according to the value of *g*(⋅), both nodes *α* and *β* would be recruited as participants to collect sensing data at the same time. However, after *α* is recruited, the necessity of recruiting *β* would decrease sharply because of the homogeneity of the sensing capabilities of participants *α* and *β*.

To solve the abovementioned problem, the matrixes of temporary data requirements **Ψ** ^*j*^ are defined as follows:

$$\begin{array}{c} \Psi^{j}=[\begin{array}{cccc} \Psi_{11}^{j} & \Psi_{12}^{j} & \cdots & \Psi_{1m}^{j}\\ \Psi_{21}^{j} & \Psi_{22}^{j} & & \\ \vdots & & \ddots & \\ \Psi_{l1}^{j} & & & \Psi_{lm}^{j} \end{array}] \end{array}$$ (13)

where $\Psi_{ik}^{j}$ denotes the temporary data requirements of task *j* at location *i* during time *k*. At the initial time, we let $\Psi_{ik}^{j}=R_{ik}^{j}$. After *α* is recruited, $\Psi_{ik}^{j}=\Psi_{ik}^{j}-\theta_{ik}^{j}\left(\alpha \right)$, and **R** ^*j*^ in Eq (12) is replaced by **Ψ** ^*j*^. Then,

$$\begin{array}{ccc} g(\alpha ) & = & \sum_{j=1}^{n}\lambda_{j}\times {∥\Psi^{j}-\Theta^{j}(\alpha )∥}_{F}\\ & = & \sum_{j=1}^{n}\lambda_{j}\times (\sum_{i=1}^{l}\sum_{k=1}^{m}{\left(\Psi_{ik}^{j}-\theta_{ik}^{j}(\alpha )\right)}^{2}) \end{array}$$ (14)

The expectations on incentives by different participants during one system duty cycle are also not similar. We let *C*_*α*_ denote the incentive requirements of *α* during one system duty cycle. The utility of *α* can be designed as follows:

$$\begin{array}{c} U_{\alpha }=\frac{g(\alpha )}{C_{\alpha }} \end{array}$$ (15)

where *U*_*α*_ denotes the sensing data amount that participant *α* is able to collect under unit incentive.

### 5.2 Optimal Heterogeneous Participant Recruitment Algorithm

Given that participants exhibit random mobility, the differences between the predicted and real trajectories result in differences between the data expectations and data collection results. Therefore, an optimal heterogeneous participant recruitment (HPR) strategy was developed to eliminate the discrepancies between data expectations and data results.

The optimization formula in Eq (7) belongs to the knapsack problem. Therefore, a greedy algorithm denoted as Algorithm 1 was designed to find solution *X* in Eq (7). The goal of Algorithm 1 is to recruit a set of participants from candidates to collect as much sensing data as possible for all the sensing tasks with limited incentives.

**Algorithm 1**: Optimal heterogeneous participant recruitment algorithm

**Input**: Position transition matrix *P*; Subareas *L*; Sensing tasks *J*; Task incentives *C*_*total*_; System duty cycle *T*; Data requirements *R*; Data obtained *D*; All candidate participants *M*; Data Sampling interval *ɛ*.

**Output**: Selected participants set *X*

1 *X* = *ϕ*;

2 **for** (*j* = 1, *j* ≤ *n*, *j*++) **do**

3  **Ψ** ^*j*^ ← **R** ^*j*^;

4 **end**

5 **while** *M* ≠ *ϕ* and *C*_*total*_ > 0 **do**

6  *α* ← arg max_*β* ∈ *M*_{*U*_*β*_};

7  *C*_*total*_ ← *C*_*total*_ − *C*_*α*_;

8  **if** *C*_*total*_ ⩾ 0 **then**

9     put *α* into *X*;

10   remove *α* from *M*;

11   **for** (*j* = 1, *j* ⩽ *n*, *j*++) **do**

12    **Ψ** ^*j*^ ← **Ψ** ^*j*^ − **ϴ** ^*j*^(*α*);

13   **end**

14  **end**

15  **else**

16   remove *α* from *M*;

17   *C*_*total*_ ← *C*_*total*_ + *C*_*α*_;

18  **end**

19 **end**

20 return *X*;

The algorithm recruits participants in loops. The participant with the highest utility is selected and paid with incentives he or she expected within one loop until all incentives are depleted or all candidates are evaluated. Algorithm 1 runs at the beginning of each system duty cycle. All the selected participants in the algorithm participate in sensing data collection in the following system duty cycle. The set of *X* selected by HPR is proven to be a feasible solution to the optimization problem in Eq (4).

The participant selection strategy runs on the server side. The time complexity of the algorithm is *O*(*n* ^2^). The time consumption of the strategy is still within milliseconds even if the total number of all available participants reaches *2000*. The computation complexity of HPR is not a restriction in the sensing task assignment phase.

## 6 Evaluation

Real vehicle GPS traces from the T-drive trajectory [33, 34] were utilized to simulate and evaluate the proposed optimal HPR strategy. The T-Drive trajectory data set contains one-week trajectories of 10,357 taxis. The total number of GPS points in this data set is approximately 15 million, and the total distance of the trajectories is 9 million kilometers. The time frequency of data sampling was set to 5 s. Taxi drivers can usually find an optimal rout to the destination based on their experience. Therefore, the T-Drive project was trying to improve the efficiency of the navigation software according to the experience from taxi drivers. On the other hand, the traces of the taxis can reflect the characters of the passengers. Since the mobility of the passengers are not completely random, the mobility of the taxies are also predictable. For example, Huang et al. [35] designed a vehicle mobility model based on the regular patterns derived from the traces of 4000 taxies in Shanghai.

**Fig 3:**
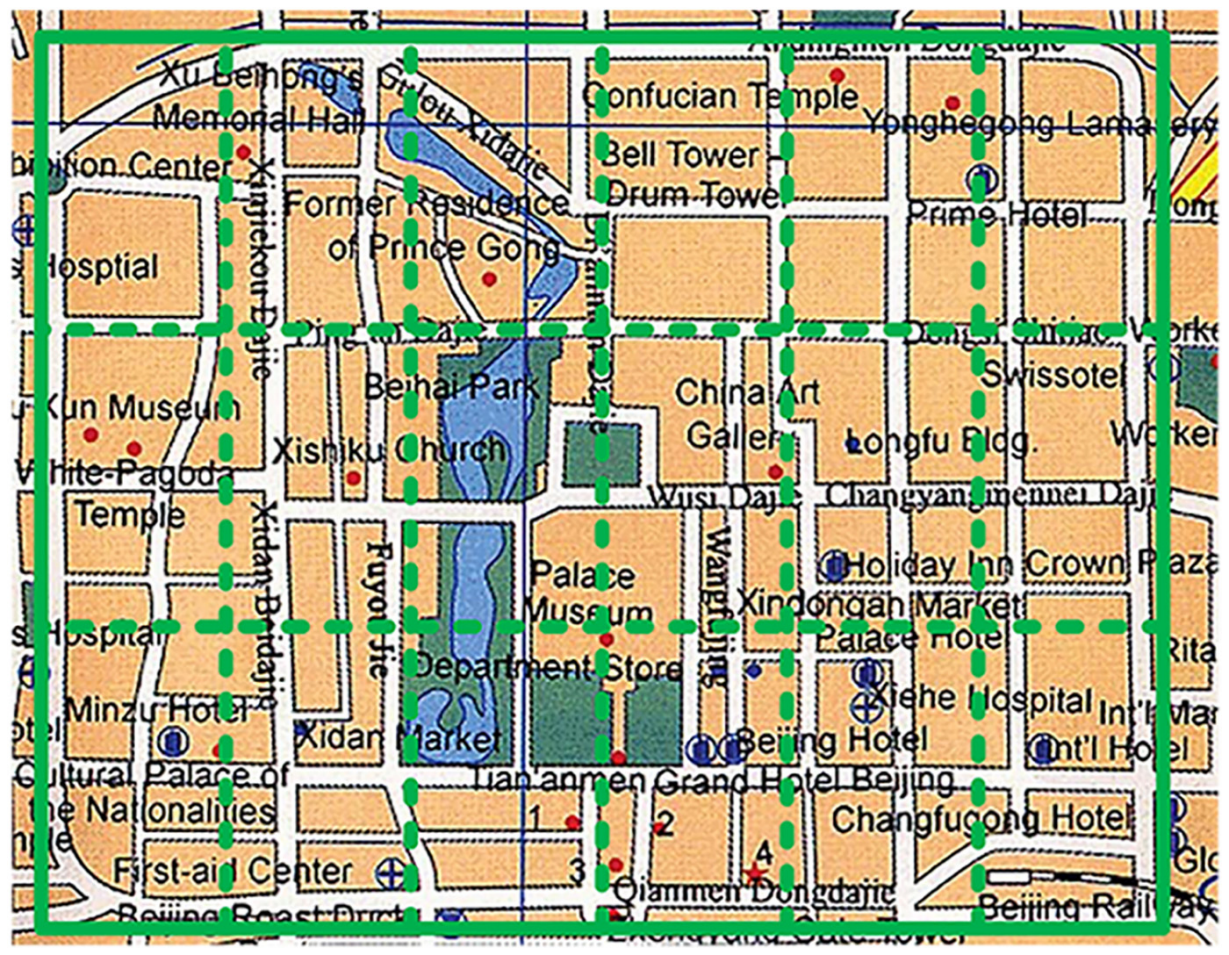
Sensing area.

In our simulation, a rectangular region around the Second Ring Road of Beijing was utilized as the target sensing area (Fig 3). Traces in the rectangular area were considered in the simulation. The 53 longest traces in the rectangular region were utilized as available vehicles, and 568 shorter traces were utilized to calculate the transition probability between subareas.

The default parameters of the experiments were set as follows. The number of sensing tasks in the rectangular region is three, and the total time of the experiment is 8 h. The rectangular region was divided into 18 subareas. The unit sensing time period was set to 60 s. The system duty cycle was set to 30 min. The data requirement of each task in each subarea in each unit sensing time period was set to 30 samplings. Three types of sensors were employed to collect data for the three tasks, and each participant has 50% possibility to be equipped with each sensor.

The incentive requests for one sampling of all available participants were random values between 0 and 2, and the average value of incentive requests was 1. Given that the sampling frequency was set to 5 s per sampling, 12 samplings could be completed by one participant in a unit sensing period. The default sensing incentive costs provided by each task were 60(= 5 × 12) for a unit sensing time period or 5 for one sampling. The total incentives for one sampling were 15; this condition means that the three tasks could afford an average of 15 participants for each time of sampling. The simulation parameters and their default values are shown in Table 2.

**Table 2:** Simulation parameters.

Parameter	Default Value
Simulation time	8 hours
Unit time	60 seconds
System duty cycle	30 minutes
Total incentives	5300
Participant number	53
Average incentive for one sampling	1
Sampling frequency	0.2 times per second

There was no participant recruit algorithm proposed for heterogeneous vehicle sensing before HPR. As a result, we compared the HPR algorithm with the random recruit (RR) method, as shown in the following evaluations. The RR method selects participants randomly at the beginning of each system duty cycle until the total incentive budget of the system duty cycle runs out. Three experiments were performed to evaluate the two participant recruitment strategies under different conditions.

### 6.1 Impact of the incentive budget

**Fig 4:**
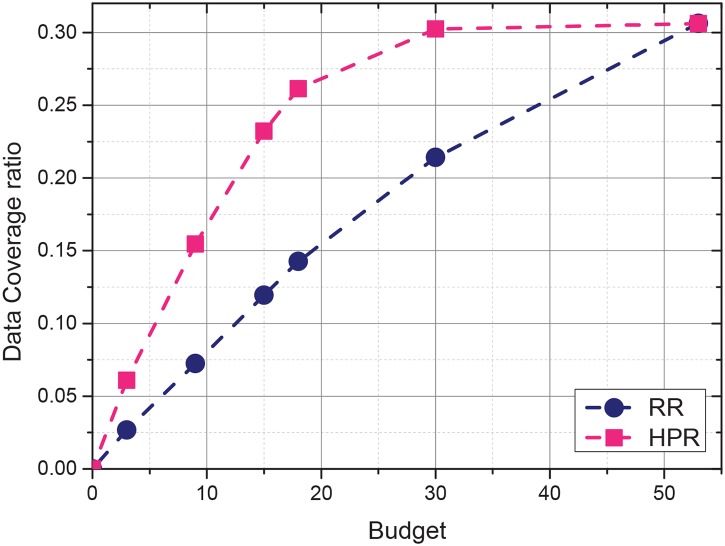
Data coverage ratio under different incentive budgets.

This subsection evaluates the impact of total incentive budget on data coverage ratio in temporal and spacial dimensions. The relative data coverage ratio is calculated by the ratio between the amount of collected data and the amount of all the data could be collected in the target sensing area. The total incentive budget is the biggest impact factor to the data coverage ratio since it determines the number of the recruited participants, which further determines the amount of sensing data can be collected. Given the following total incentives for one system duty cycle, {3,6,9,12,15,18,30,45,53}, the sensing data coverage ratio results are given in Fig 4. The collected data could not match all the data requirements, even when 53 available vehicles were all selected. The reason is that the traces are not uniformly distributed in spatial and temporal spaces.

Fig 4. shows the impact of the total incentive budget on the collected sensing data coverage ratio. The experiment was executed 10 times, and the results in Fig 4, are the average values from the 10 executions. Fig 4. shows that the sensing data coverage ratios of the two strategies increase with the increase in total incentive budget. If the total budget is small, the coverage ratio of HPR increases faster than that of RR. When the total budget is small, the number of participant vehicles that could be recruited is constrained. However, the HPR strategy selects participants with high sensing utilities. Therefore, the HPR strategy can achieve a high sensing data coverage ratio. Nevertheless, the total amount of participants that could be recruited is limited (i.e., 53). The data coverage ratios of the two strategies are close when all participating vehicles could be recruited to the system.

The relation between the incentive budget and data coverage ratio of RR is approximately linear. However, the data ratio gained by HPR increases rapidly before the total incentive budget reaches 35. In the experiment, the coverage ratio of the collected sensing data is 31.5% when all the 53 participants are recruited to participate, while the coverage ratio is 26.9% when only 18 participants could be recruited under limited budget. In other words, HPR collects most (0.315/0.269 = 85.4%) of the available sensing data with 18/53 = 34% incentive cost. The data coverage ratio of HPR increases gradually at a later period but remains larger than that of RR. Thus, with a limited incentive budget, HPR can maximize the collected sensing data coverage ratio by preferentially recruiting the most efficient participants.

### 6.2 Impact of the required data volumes

**Fig 5:**
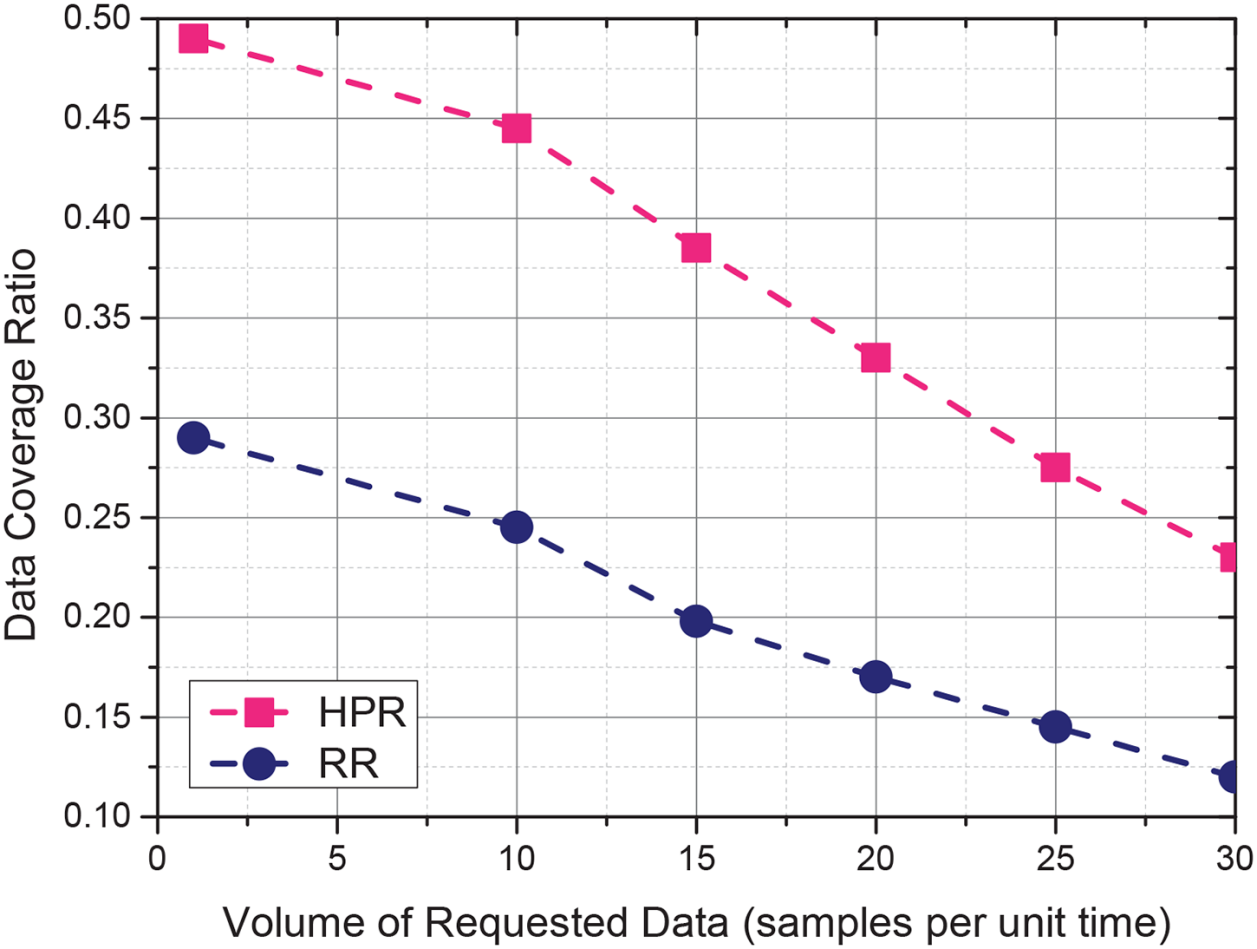
Impact of required data volumes.

Different participatory sensing scenarios request different volumes of data in the target sensing area. This experiment is designed to test the sensing data coverage ratio under different volumes of requested sensing data. The total incentive budget is 18. We change the volumes of the required sensing data. for each volume, the experiment is repeated for 10 times and the average collected sensing data ratios are recorded. The experiment result is shown in Fig 5. When the requested data volume is small, both RR and HPR achieve high data coverage ratios. Throughout all the requested volumes, HPR behaves better than RR.

Different participatory sensing scenarios require different volumes of data in the target sensing area. An experiment was designed to test the sensing data coverage ratio under different volumes of required sensing data. The total incentive budget is 18. We changed the volumes of the required sensing data. For each volume, the experiment was repeated 10 times, and the average collected sensing data ratios were recorded. The experiment result is shown in Fig 5. When the required data volume is small, both RR and HPR achieve high data coverage ratios. HPR performs better than RR in all the required volumes.

### 6.3 Fluctuations of the sensing results

Due to the mobility of the vehicles and the recruitment of the vehicles are random, the coverage ratio of the selected data fluctuate over time. The sensing data coverage ratios of 8 simulation hours are evaluated in this experiment. The total incentive budget is set to 30. Each simulation is executed for 10 times, and the errors of the results are investigated.

The coverage ratio of the selected data fluctuates over time because of the mobility of vehicles and the random recruitment process of vehicles. The sensing data coverage ratios of 8 h of simulation were evaluated in this experiment. The total incentive budget was set to 30. Each simulation was executed 10 times, and the errors of the results were investigated.

**Fig 6:**
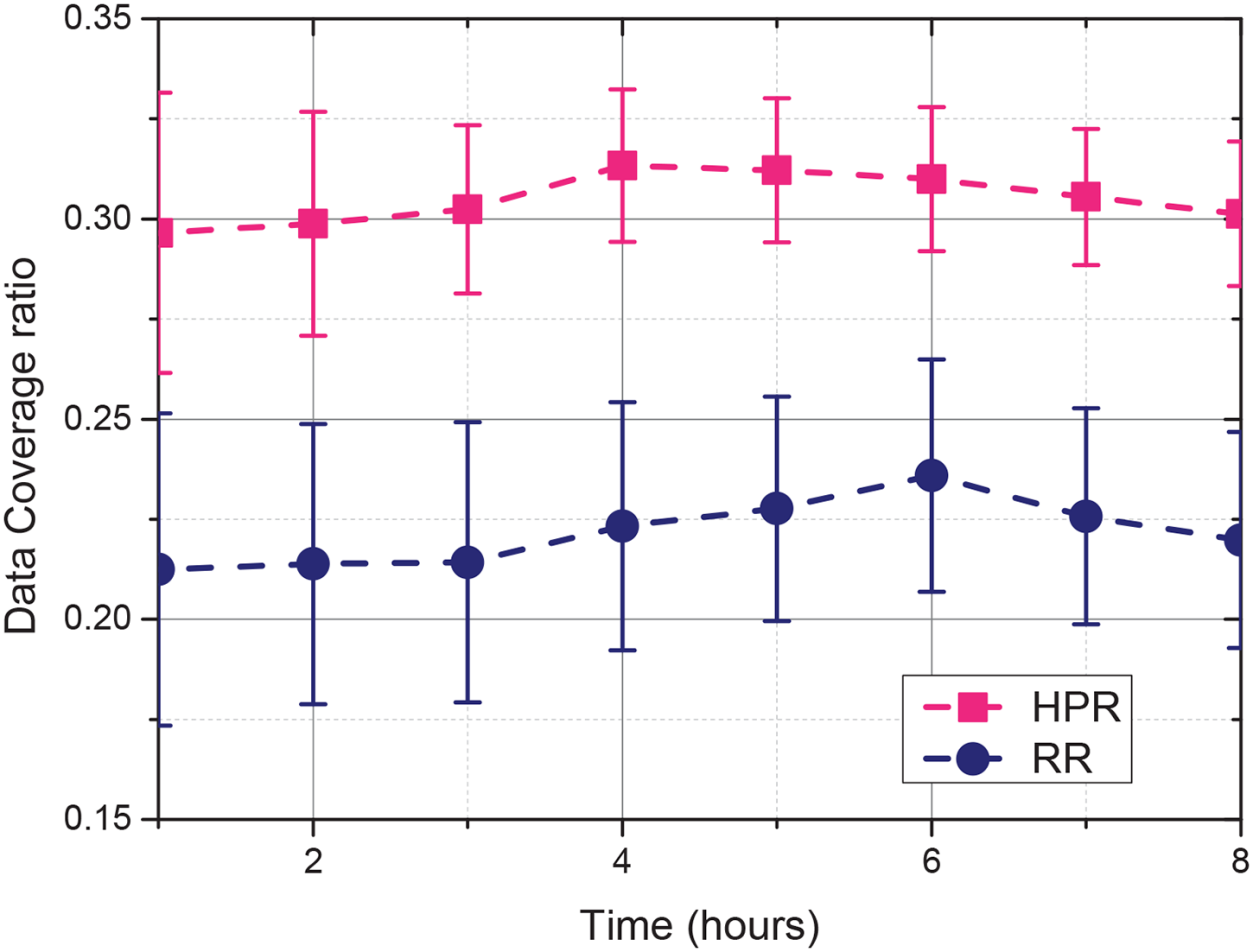
Fluctuations in the sensing results.

As shown in Fig 6. with the same total incentive budget, the sensing data coverage ratio of HPR is higher than that of RR. The coverage ratios of the two recruitment strategies fluctuate in a limited range. Given that HPR predicts the mobility traces of the recruited vehicles, the amplitude of the fluctuations of HPR is smaller than that in RR. Therefore, the stability of the quality of the sensing data collected by HPR is higher than that collected by RR.

### 6.4 Distribution of sensing interfaces

**Fig 7:**
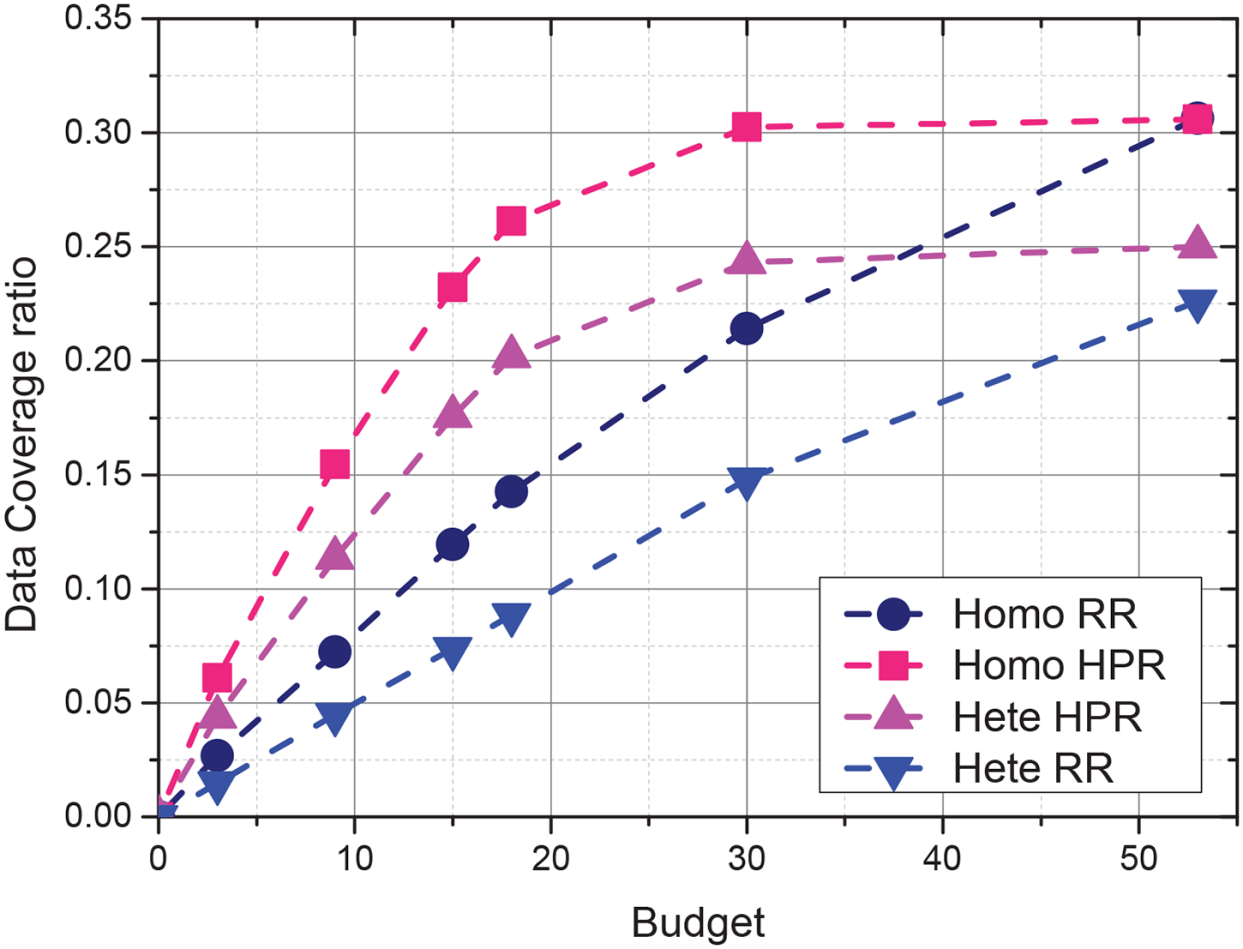
Impact of sensing interface distribution on sensing data coverage.

The relationship between the sensing data coverage ratio and the incentive budget were evaluated with different sensing interface distributions. The distribution of the three sensing interfaces of the vehicles was set to homogeneous (50%,50%,50%) and heterogeneous (20%,50%,80%). As shown in Fig 7. the evolution trends of the vehicle recruitment strategies are similar. However, the coverage ratio obtained by the heterogeneous sensing vehicles is 15% lower than obtained by the homogeneous vehicles. The reason is that sensing data with a rare sensing interface distribution are difficult to collect, whereas sensing data with widely distributed sensing interfaces are usually superfluous. HPR is less affected by the distribution of the sensing interfaces than RR because HPR selects more efficient vehicles and can compensate for the heterogeneous distribution of sensing interfaces.

### 6.5 Impact of different incentives provided by tasks

The goal of this experiment is to determine the impact of different incentives provided by tasks. The previous experiments assumed that all three tasks are equally important and have the same incentives. However, the importance of different tasks can differ, as shown by the differences in task incentives.

**Fig 8:**
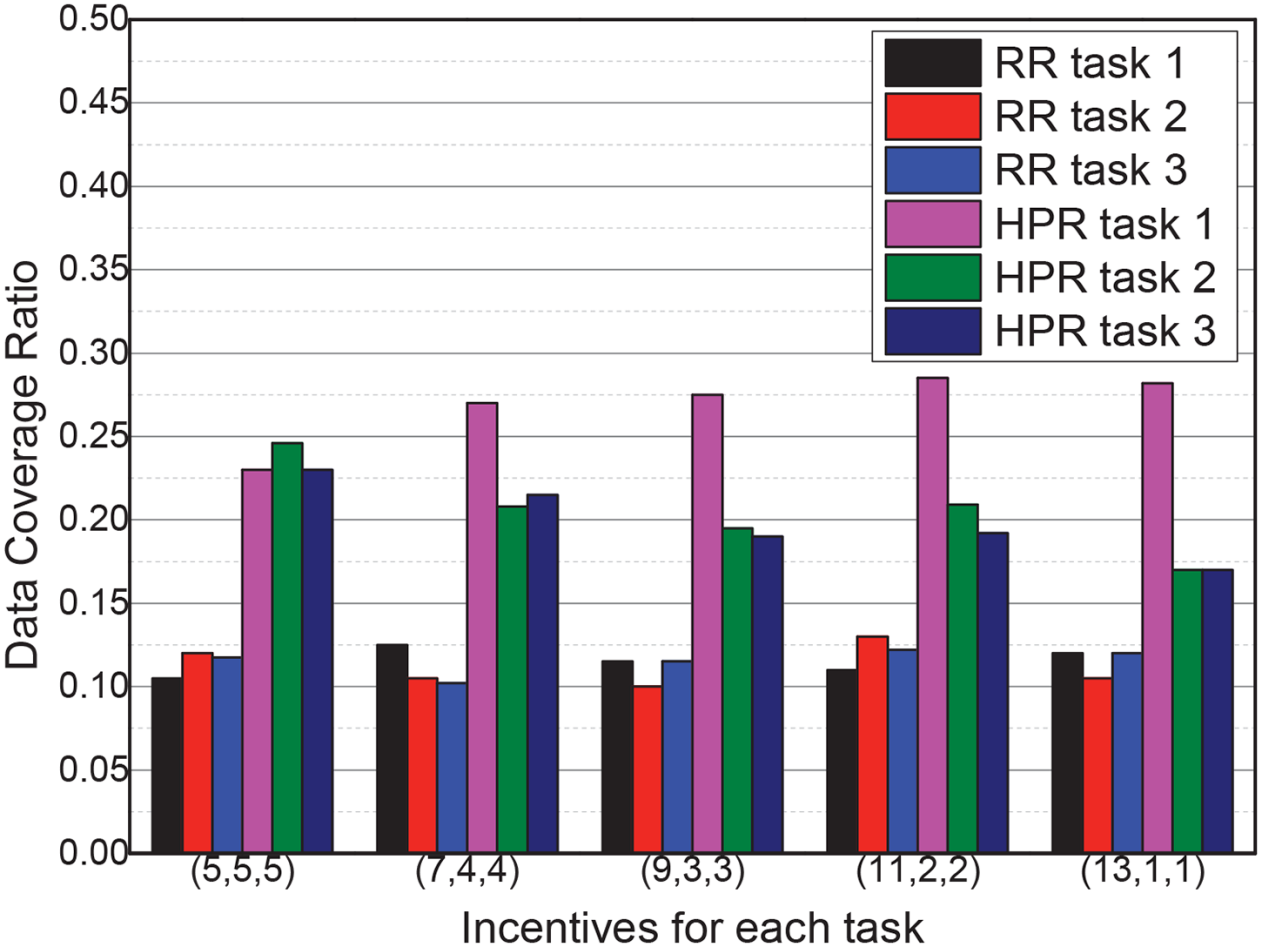
Impact of different task incentives in RR and HPR.

In this experiment, the total incentive of all three tasks was the same (15 per sampling), whereas the incentives of each task were changed from (5,5,5) to (13,1,1) gradually. The data coverage ratios of each task were recorded, and the results are shown in Fig 8. When the incentives change between tasks, the data coverage ratios of RR remain constant, whereas the data coverage ratios of HPR change significantly. This experiment shows that tasks with higher incentives can obtain more data through the proposed dynamic participant selection method.

### 6.6 Impact of different task significance

This experiment evaluates the impact of different distributions of the task significance coefficient factor. In the previous experiments, {*λ*_1_, *λ*_2_, *λ*_3_} are set to the same value {0.33,0.33,0.33}, and all the sensing tasks have the same significance. However, in some scenarios, sensing tasks may have different significance. The significance property of the sensing tasks in HPR is indicated by *λ*, whose distribution will affect the value of the utility of each sensing node.

**Fig 9:**
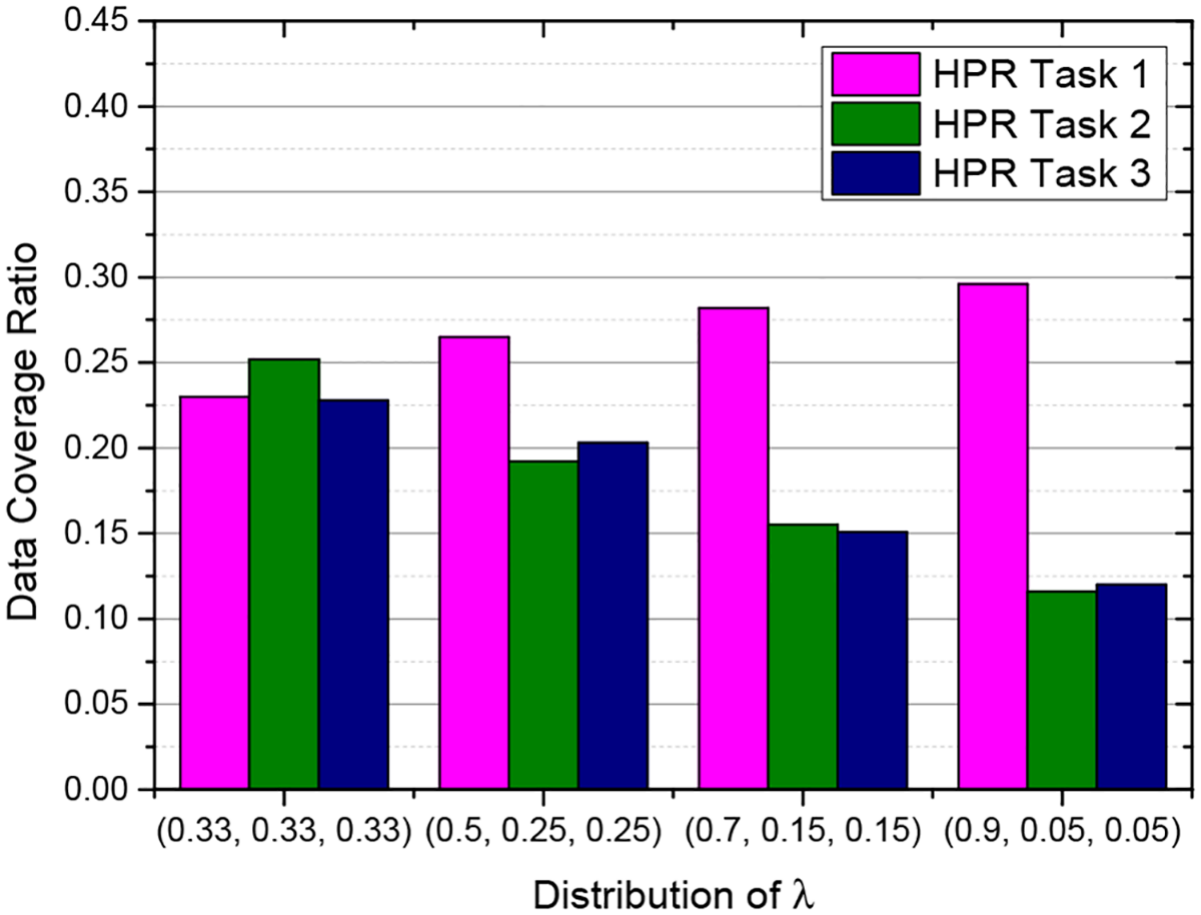
Impact of different task significance in HPR.

In this experiment, {*λ*_1_, *λ*_2_, *λ*_3_} are set to {0.33,0.33,0.33}, {0.5,0.25,0.25}, {0.7,0.15,0.15}, {0.9,0.05,0.05}, respectively. As shown in Fig 9, the coverage ratio of the sensing task is low, when the corresponding significance coefficient is small. Especially, when the coefficient of task 2 and task 3 are set to 0.05, the coverage ratio of the both reduced to the level near that of the RR. It means that the HPR will degrade into RR, if the coefficient *λ* is set to 0. On the other hand, if the sensing task is significant, the coefficient of the task could be set to a big value, and as a result, the coverage ratio of the sensing task would be high.

## 7 Conclusion

Owing to the limitation of the total incentive and the heterogeneity of vehicular participants in terms of sensing capabilities, trajectories, and incentive expectations, a group of vehicles should be recruited to participate in sensing data collection to collect comprehensive and fresh sensing data for the sensing system. Therefore, an optimal heterogeneous participant recruitment strategy was developed. The models of the requirements of sensing data, the collected sensing data, and the expected sensing data were first defined. blackA optimization formula was established to model the participant recruitment problem. A participant utility analysis scheme was built based on the sensing and mobility features of the participants. Utility was employed to evaluate the incentive efficiency of the sensing participants based on the ratio of their expected data contributions to their incentive requirements. A dynamic participant selection strategy was then designed. The participants were selected through a greedy algorithm that evaluates the efficiencies of the participants based on their utility. The real trace-driven simulations show that the proposed strategy can collect 85.4% of available sensing data with 34% incentive budget.
